# How to prevent dislocation after revision total hip arthroplasty: a systematic review of the risk factors and a focus on treatment options

**DOI:** 10.1186/s10195-018-0510-2

**Published:** 2018-09-10

**Authors:** C. Faldini, N. Stefanini, D. Fenga, E. M. Neonakis, F. Perna, A. Mazzotti, F. Pilla, I. K. Triantafyllopoulos, F. Traina

**Affiliations:** 1 1st Orthopaedic and Traumatologic Clinic, IRCCS Istituto Ortopedico Rizzoli, Via Giulio Cesare Pupilli 1, 40136 Bologna, Italy; 2 Department of Biomedical and Dental Sciences and Morphofunctional Imaging, University Hospital “G.Martino”, Messina, Italy; 3 Agia Sofia General Children’s Hospital, Athens, Greece; 4 Medical School, University of Athens, Athens, Greece

**Keywords:** Revision, Dislocation, Hip arthroplasty, Failure, Risk factors, Surgical treatment

## Abstract

**Background:**

Dislocation represents the most common complication after revision total hip arthroplasty (rTHA). Understanding risk factors for dislocation has a great clinical relevance for every hip surgeon in order to consider all surgical options for effective planning. The aim of this systematic review was to answer two main questions—(1) what are the risk factors for instability after rTHA? and (2) what are the best preoperative assessments and surgical options to avoid dislocation after rTHA?

**Materials and methods:**

Scientific databases were accessed to identify papers dealing with prevention and treatment of dislocation after rTHA. We performed a search using the keywords ‘revision hip arthroplasty’ and ‘dislocation’, ‘instability’, ‘outcome’, ‘failure’, ‘treatment’. After removal of duplicates and exclusion of works published in different languages, 33 articles were reviewed completely.

**Results:**

Risk factors were analysed in order to establish the most relevant and evidence-based treatments available in the current literature.

**Conclusions:**

The risk of dislocation after rTHA can be reduced using some precautions inferred from the literature. The use of a larger femoral and acetabular component, elevated rim liner and dual mobility implants can significantly reduce the risk of dislocation after rTHA. However, care must be taken regarding patient-related risk factors since these cannot be addressed and modified. Hence, a complete evaluation of risk factors should be performed for each patient and procedure before starting rTHA.

## Introduction

Revision total hip arthroplasty (rTHA) is a growing surgical procedure which is likely to increase further in the future as a consequence of the expected rise in primary hip arthroplasty procedures [1]. Dislocation is the main cause of failure after rTHA [2], compromising both long-term function of the joint and patient satisfaction and increasing the risk of successive revision surgeries.

The reported incidence of dislocation after rTHA is as high as 39% [3] and in some cases re-revision surgery is an inevitable procedure. According to the literature, risk factors for dislocation after rTHA can be divided into patient-related and procedure-related factors [4]; however, they are multifactorial and are not as well understood as the risk factors for instability after primary THA. A clear strategy on how to prevent them is not yet available in the literature. The aim of this study is to assess the risk factors and mechanisms of dislocation after rTHA inferred from a systematic review of the available literature in order to identify a possible strategy to prevent dislocation.

The research raised two important questions—(1) what are the risk factors related to instability after rTHA? and (2) what are the best preoperative assessments and surgical options to avoid dislocation after rTHA?

## Materials and methods

We performed a search using the keywords ‘revision hip arthroplasty’ in combination with ‘dislocation’, ‘instability’, ‘outcome’, ‘failure’ and ‘treatment’. English journal articles on these items were searched using PubMed (http://www.ncbi.nlm.nih.gov/sites/entrez/); Ovid (http://www.ovid.com/); Cochrane Reviews (http://www.cochrane.org/reviews/) and Google Scholar databases. No limit regarding the year of publication was set. All journals were considered. After removing duplicates, the research yielded 542 results, including case reports, letters, communications, and prospective and retrospective studies.

All papers investigating risk factors, prevention and/or treatment for dislocation after rTHA for any cause in any population group were included in this study.

Exclusion criteria were studies on dislocation after primary total hip replacement, studies on dislocation after trauma, and studies on dislocation after THA secondary to bone tumors and metastasis. Study design was also an exclusion criteria as case reports, letters and communications were not considered in the study.

Two authors read the abstracts and excluded the articles that were unrelated to the topic of the study. In case of doubt regarding inclusion of an article, the senior author made the final decision. Based on title, abstract and study design 397 articles were excluded. Exclusion was made because of study design for 281 articles and because the topic of the study was not related to dislocation after rTHA in 116 articles. The full text version of the remaining 145 articles was obtained and the reference lists were screened to provide a further 9 articles related to the topic. The content of the 154 resulting articles were discussed by all the co-authors and 33 of the studies were considered eligible for inclusion in this review.

**Fig. 1:**
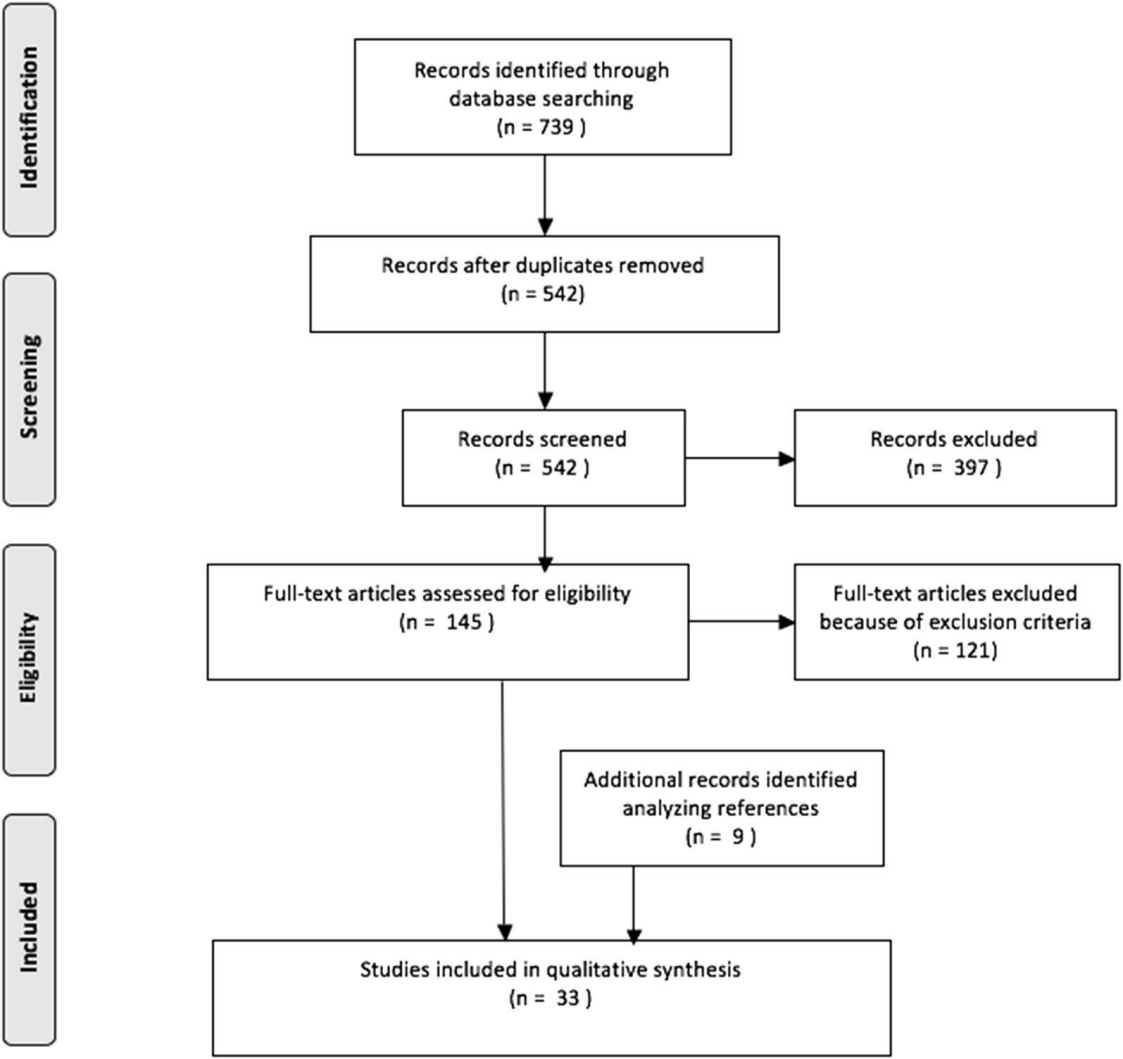
Flow chart of selection procedure of the reviewed papers

A PRISMA flow chart was used to summarize the selection procedure of the reviewed papers (Fig. 1).

The modified Coleman methodology score (MCMS) was used to assess the quality of the included studies. This 15-item score is used for assessment of study quality, with scores ranging from 0–100 (studies with a score < 55 are considered poor quality) [5].

## Results and discussion

The research included 33 papers for review. Among them, 15 were eligible for a systematic review and analysis of risk factors for dislocation after rTHA. The remaining 18 articles were inherent to the treatment options and were presented in a narrative way.

Two of the authors evaluated the level of evidence and analyzed the results. Of the 15 articles regarding risk factors, 12 were Level IV (80%) and 2 were Level III (13.3%). Only one (6.6%) Level I study was identified. The MCMS was < 55 for all included studies except one.

Risk factors related to dislocation after rTHA could be divided into patient-related and procedure-related factors [4] as shown in Tables 1 and 2, whereas Table 3 shows the characteristics of the study.

**Table 1:** Patient-related risk factors

Authors	Previous revision surgeries	Abductor deficiency	History of at least one episode of instability	ONFH	Severe acetabular and femoral bone loss	Greater age
Alberton et al. [12]		X				
Khatod et al. [6]	X					
Springer et al. [2]	X					
Carter et al. [7]	X	X				
Cogan et al. [8]	X					
Kosashvili et al. [9]	X					
Wetters et al. [4]		X	X		X	− (ns)
Jo et al. [10]	X					
Yoshimoto et al. [11]	X	X	X	X		− (ns)
Yoshimoto et al. [13]		X		X		+

**Table 2:** Procedure-related risk factors

Authors	Small femoral head diameter	Single-component revision	Use of standard rim liner
Alberton et al. [12]	X		X
Khatod et al. [6]			
Sah et al. [14]	X		
Hummel et al. [15]	X		
Carter et al. [7]	X		
Kosashvili et al. [9]	X		
Garbuz et al. [16]	X		
Wetters et al. [4]	X		
Kosashvili et al. [17]		X	
Jo et al. [10]	X		
Stedman et al. [18]		X	
Yoshimoto et al. [13]	X

**Table 3:** Characteristics of the studies

Authors	Population (no. of hips)	Follow-up (years)	Level of evidence
Alberton et al. [12]	1548	Mean 8.1	Level IV
Khatod et al. [6]	277	Minimum 1	Level III
Sah et al. [14]	204	Mean 4.9	Level IV
Hummel et al. [15]	242	Mean 2.4	Level IV
Springer et al. [2]	1100	Mean 6	Level IV
Carter et al. [7]	156	Mean 5.6	Level III
Cogan et al. [8]	61	Mean 2.4	Level IV
Kosashvili et al. [9]	749	Mean 13.2	Level IV
Garbuz et al. [16]	184	Mean 5	Level I
Wetters et al. [4]	1152	Mean 2	Level IV
Kosashvili et al. [17]	749	Mean 13.2	Level IV
Jo et al. [10]	539	Mean 5.5	Level IV
Yoshimoto et al. [11]	178	Mean 6.7	Level IV
Stedman et al. [18]	187	Mean 7.6	Level IV
Yoshimoto et al. [13]	88	Mean 4.4	Level IV

Patient-related risk factors include:

1. Number of previous revision surgeries;
2. Abductor muscles deficiency/trochanteric non-union;
3. History of at least one episode of instability before revision surgery;
4. Osteonecrosis of the femoral head (ONFH);
5. Severe acetabular and femoral bone loss;
6. Age.

Procedure-related risk factors include:

1. Small femoral head diameter;
2. Single-component revision;
3. Use of a standard rim liner.

### Patient-related risk factors

#### Number of previous hip surgeries [2, 6–11]

The number of revision surgeries is directly related to dislocation rate. Carter et al. [7] found a dislocation rate of 32% in cases of repeated rTHA compared to 15% in cases of first-time rTHA. Similarly, Kosashvili et al. [9] found increased dislocation rates of 35, 46 and 383% after second, third and fourth or more rTHA when compared with first-time rTHA. Moreover, Wetters et al. [4] indicated that patients undergoing revision for instability after a previous rTHA had a 19% (25 of 129) rate of dislocation after the procedure. Recently, Jo et al. [10] found an increased hazard ratio for dislocation after rTHA (1.46) in patients with a history of ≥ 2 previous hip surgeries.

#### Abductor muscles deficiency [4, 7, 11–13]

Abductors at the time of revision surgery are estimated and classified as either intact or deficient. The latter is due to abductor post-surgical trochanteric non-union, detachment or myopathy [4]. Alberton et al. [12] reported 7 cases of dislocation in 9 trochanteric non-unions after rTHA from 1548 rTHAs in 1405 patients. Wetters et al. [4], in a retrospective review, identified abductor deficiency as a stronger predictor risk factor for dislocation with an odds ratio (OR) of 2672. Recently, Yoshimoto et al. [13] reported a re-dislocation rate of 18.2% after rTHA (16 re-dislocations in 88 rTHAs), finding a correlation between the rate of re-dislocation and joint laxity, including necrosis of the gluteus muscles, which was identified in 7 of 16 hips.

#### History of at least one episode of instability before revision surgery [4, 11]

rTHA performed in a dislocating hip or in patients suffering from previous dislocation has a higher risk of further dislocations.

Wetters et al. [4] found that an unstable implant presented a history of instability in 46% of cases (52 of 113 patients) compared to a stable implant which presented a previous history of instability in 24.2% of cases (251 of 1039 patients).

Similar results were reported by Yoshimoto et al. [11] who found that 20% of patients who had dislocation after rTHA (16 of 178) had a previous dislocation before revision surgery, which was significantly higher than 5.4% patients who did not have a dislocation after rTHA.

#### Osteonecrosis of the femoral head [11, 13]

Yoshimoto et al. found that a diagnosis ONFH at the time of primary surgery was also an independent risk factor for dislocation after rTHA [11]. The authors suggest that the dislocation could have been induced by the reduced soft-tissue stiffness, which can cause a higher range of motion (ROM) [13], in addition to the primary causes that could have led to osteonecrosis (alcohol and use of corticosteroid).

#### Severe acetabular and femoral bone loss [4]

In cases of severe bone loss, the revision procedure is complicated and troublesome and proper component orientation is not always possible to achieve leading to an increased dislocation rate. Wetters et al. [4] stated that acetabular defect complexity (Paprosky classification of ≥ 3) represents a risk factor because of the difficulties in restoring the centre of rotation of the hip. Proper cup position may also be difficult in these patients due to the altered anatomy.

#### Age [4, 10, 11, 13]

The effects of age on dislocation have not been fully understood in the available literature. Wetters et al. [4] found that younger patients present a higher risk of dislocation after rTHA, probably due to enhanced activity levels. Patients who had a dislocation subsequent to rTHA were an average of 3 years younger (62 vs 65) than patient who did not dislocate; however, these values did not reach statistical significance.

Conversely, Jo et al. [10] found no differences in dislocation rate after rTHA per 10-year increment; however, again without statistically significant differences.

Furthermore, two multicentre studies [11, 13] found a statistically significant 2.9-fold rise in the risk of dislocation after rTHA for each 10-year age increase. This was related to decreased muscle strength and the increased risk of falling [11].

### Procedure-related risk factors

#### Small femoral head diameter [4, 7, 9, 10, 12–16]

The size of the femoral head component has been widely accepted as a risk factor for dislocation after rTHA with an increased risk in cases of smaller femoral head diameter.

Alberton et al. [12] found a significant increase in the number of unstable hips after rTHA using a 22-mm head compared to a 26-mm head or larger sizes. Similarly, Wetters et al. [4] found that unstable implants present more frequently with a smaller head size (mean 34.2 mm) compared to stable implants (mean 36.0 mm). Garbuz et al. [16] reported a dislocation rate of 1.1% in the group with a larger head diameter (36 or 40 mm) and 8.7% in cases with a smaller head diameter (32 mm). Recently, Yoshimoto et al. [13] found that 12 of 16 patient (75%) who had a dislocation after rTHA, were revised using a femoral head < 32 mm and that 30 of 72 patients (42.3%) who did not dislocate were revised using a larger femoral head.

#### Single-component revision [17, 18]

In cases of rTHA, the procedure could involve one or both components. A higher dislocation rate was found in the literature in cases of single-component revision. Kosashvili et al. [17] found a significantly higher dislocation rate (10.28%) in cases of isolated acetabular revision compared to revision of both components or femoral stem-alone revision (6.61 and 0%, respectively); however, no statistically significant differences were found.

Conversely, Jo et al. found an increased rate of dislocation after rTHA when the acetabular cup is retained. Other authors [2, 4, 8, 10–12] found a trend toward an increased dislocation rate when rTHA results in combined component malposition. However, none of those studies reached statistical significance. We did not retrieve any study evaluating the relationship between the combined anteversion techniques and rTHA. Since the combined anteversion technique is an important topic for stability in primary hip arthroplasties [19–22], further studies addressing this argument could be useful.

#### Use of standard liners in revision surgery [9, 12]

Alberton et al. [12] reported a 3.8% dislocation rate after acetabular components revised with an elevated rim liner compared to 8.4% of dislocations after acetabular components revised with a standard rim. Similar results were achieved by Kosashvili et al. [9] although were not statistically significant.

### Preoperative assessment and treatment options

The aim of surgery, as for primary hip arthroplasty, is the restoration of the original hip biomechanics, similar to contralateral hip [23].

A preoperative templating is always recommended in order to obtain effective position and orientation components. Abductor muscle deficiency should be assessed while planning rTHA. A computed tomography (CT) scan of the hip should be performed routinely to plan rTHA in order to assess the bone stock, and muscle tissue quality should also be assessed. If the CT scan is not satisfactory, magnetic resonance imaging should be performed for further evaluation of possible abductor muscle deficiency.

A surgical approach is a significant surgical choice both for primary [24, 25] and revision [26–28] procedures, and should be based on several factors, such as previous approach, soft tissue and bone condition, and surgeon experience.

The type of approach may influence the risk of dislocation. A study by Hailer et al. [29] of 78,098 THAs extracted from the Swedish Hip Arthroplasty Register and considering both primary and revision surgery between 2005 and 2010, reported 399 hip revision procedures due to dislocation; the posterior approach was associated with a higher risk of dislocation compared to the direct lateral approach and anterior approach.

However, no specific articles regarding the risk of dislocation secondary to the approach used in cases of rTHA are reported in the current literature.

Proper muscular tension should always be evaluated, especially when dealing with abductor muscle deficiency. This could be achieved by modifying the femoral offset, either by medializing the femoral centre of rotation or by using a lateralized stem or bigger femoral head.

According to the literature, regardless of the failed prosthetic component, the ideal solution should be revision of both the femoral and the acetabular side, thus being able to choose either best positioning or restore correct offset [17, 18].

### Implant options

#### Larger femoral and acetabular components

Based on the retrieved literature, it is advisable to use a 36-mm head diameter or larger when performing rTHA.

A jumbo femoral head has a maximal head to neck ratio and this minimizes implant impingement [30].

Larger heads need to be coupled with larger acetabular components; however, the latter potentially produce impingement with iliopsoas muscle or tendon, giving anterior hip pain. When using an increased head size, the polyethylene liner thins to accommodate it, but only to a point (the liner has a certain minimum thickness). Therefore, femoral heads > 38 mm require larger acetabular components so that a liner of at least minimum permissible thickness can be used. Theoretically, the surgeon can decide to ream for a larger cup to accommodate the larger head, creating more acetabular bone loss.

In cases of rTHA with moderate to extensive acetabular bone loss, a jumbo acetabular component represents an effective solution. Several studies reported a high rate of success with up to 90% 10-year survival. However, long-term studies have shown late loosening of ‘first-generation’ porous surfaces and wear associated with periprosthetic osteolysis, caused by standard polyethylene liners. ‘New generation’ polyethylene, as highly X-linked polyethylene and vitamin E-doped polyethylene reduce the rate of wear [31, 32].

In addition, use of larger metal heads have raised concerns regarding potential adverse local tissue reactions (ALTRs), secondary to corrosion and metal release at the head–neck taper junction. Increasing the head size generates larger torsional forces at the trunnion–head junction, and significantly increases the maximal principal stress in the neck medial area, regardless of the material used for the head. These torsional forces enhance tribocorrosion and could lead to ALTRs.

Therefore, the use of large diameter femoral heads is advisable because the biomechanical and clinical data support the finding of increased stability and increased ROM advantages despite the increased bearing surface wear, which may affect implant longevity.

#### Elevated rim liner

When approaching revision surgery, the liner design must be taken into consideration. An elevated rim increases the area of contact with the femoral head and theoretically guarantees a higher ROM before the head dislocates.

However, a not well-positioned elevated rim liner may increase impingement against the neck of the prosthesis at high degrees of extension/flexion and internal/external rotation, and this could counteract hip stability.

#### Dual mobility cups

Dual mobility cups are widely reported to reduce the risk of dislocation after rTHA [33–40]. Dual mobility cups are designed with a 22- or 28-mm diameter femoral head component moving inside a larger polyethylene liner. The liner moves freely in a metal shell with an inner diameter corresponding to the outer diameter of the polyethylene liner. Dual mobility components have been extensively used in Europe for > 25 years [33, 34, 41, 42]. They provide greater ROM and jump distance than a single femoral head. This is due to the additional articular surface. Furthermore, a metal dual mobility component could be cemented into a well-fixed retained acetabular shell.

Some concerns regarding dual mobility implants still remain. Some implants in the past showed intraprosthetic dislocation [43–45], a specific way of failure due to dislocation between the small femoral head and the mobile polyethylene liner. The dislocation seems to be related to the excessive motion granted by dual mobility implants that may result in impingement of the femoral neck or of the femoral component itself against the large outer polyethylene bearing, leading to polyethylene wear and loss of its inner retentive function.

An additional concern of dual mobility components is whether postoperative motion will continue to occur at both articulations or only at the polyethylene–metal interface.

Despite these issues, dual mobility has a great utility in rTHA. The design provides a low risk of recurrent instability without increasing mechanical complications, particularly when compared with constrained or tripolar cups; namely, a dual mobility liner seems to decrease the risk of dislocation without increasing the risk of loosening [39]. Dual mobility cups also have the advantage of decreasing the need for stem exchange in complex revisions, as they can be used with 22- or 28-mm heads, especially if the head–neck junction is properly designed to interact with dual mobility components. This is shown in several studies in which dual mobility components were used specifically to treat recurrent dislocation [35–38]. Furthermore, dual mobility implants have also shown good reliability in unstable hemiarthroplasty revision converted to THA [40].

### Salvage procedure

#### Constrained bearing insert

A constrained bearing insert represents another option in cases of multiple failures for dislocation [46, 47]. A constrained bearing insert consists of a polyethylene liner, which includes a 22- or 28-mm femoral head in the inner-diameter concave surface with a locking ring. This liner is inserted in a polished CoCr shell. The shell articulates with another polyethylene liner (the outer bearing) that can be inserted into a standard acetabular shell. However, some studies report a high rate of impingement between the femoral neck and acetabular cup and reduced ROM when a constrained liner is used [47, 48].

Similarly, Jo et al. [10] reported that the use of a constrained liner could be protective against re-dislocation but was not associated with a lower re-revision rate.

#### Girdlestone procedure

In cases of multiple failures, with possible deteriorating health conditions of the patient, surgical options could be limited. An excision arthroplasty of the hip, also known as a Girdlestone procedure [49], should be taken into account despite the severe limb shortening and limp.

## Conclusion

Although many revision techniques have been proposed, dislocation after rTHA still represents a major problem for the orthopaedic surgeon.

This study has some limitations, mainly due to the low level of evidence of the papers available on this topic which did not provide clear guidelines; however, this could represent a helpful starting point from which to address further research on this relevant topic.

Patients should be given advice regarding the risk of dislocation, and patients at a higher risk, such as those with abductor deficiency, a history of instability, and complex acetabular defect, should be educated regarding their condition. Surgeons performing revisions must identify patients at risk and consider all options in order to minimize the risk of dislocation. A preoperative templating is mandatory in order to obtain effective component orientation.

Surgeons must strive to use larger prosthetic components, when acetabular and femoral bone stock allows such a procedure. When moderate to extensive acetabular bone loss is assessed, rTHA could be effectively performed using larger prosthetic components.

Dual mobility components have been used in Europe for > 25 years and are particularly suitable for recurrent dislocation.

Use of constrained bearing inserts should be considered as a salvage procedure, since they reduce the rate of dislocation, but are not effective in reducing the rate of re-revision.

In cases of failure of the aforementioned options, Girdlestone excision arthroplasty represents the ultimate solution.

## Abbreviations

rTHA: revision total hip arthroplasty
THA: total hip arthroplasty
ONFH: osteonecrosis of the femoral head
OR: odds ratio
ALTR: adverse local tissue reactions
ROM: range of movement
